# Opioid Overdose Simulation in Medical Student Education

**DOI:** 10.5070/M5.52230

**Published:** 2026-01-31

**Authors:** James Mangano, Matthew J Sarsfield, Hannah Charland, Jennifer Campoli, Martin Kim, Amber Gray

**Affiliations:** * SUNY Upstate Medical University, Department of Emergency Medicine, Syracuse, NY

## Abstract

**Audience:**

The target audience for this simulation is third-year medical students, specifically those in an emergency medicine clerkship.

**Introduction:**

This topic is critically important in emergency medicine due to the ongoing opioid epidemic, which has led to a dramatic rise in overdose cases and deaths across the United States. Overdose deaths involving opioids numbered nearly 50,000 in 2019, a nearly six-fold increase since 1999.^1^ Over 70% of drug overdose deaths in 2019 involved opioids. Emergency department visits for opioid overdoses rose 30% from 2016 to 2017 in all parts of the United States. 2 Emergency departments often serve as the front line in treating opioid overdoses, where rapid recognition and timely administration of Naloxone can be lifesaving. Training medical students to recognize and manage opioid overdoses is essential to prepare them for real-world scenarios, ensuring they are equipped with the skills and confidence to respond effectively in emergencies. Educating future healthcare providers on this topic could ultimately reduce opioid-related mortality and improve patient outcomes in these high-stakes situations.

**Educational Objectives:**

By the end of the simulation session, learners will be able to: 1) accurately identify the three key clinical signs of opioid overdose (respiratory depression, pinpoint pupils, unresponsiveness), 2) identify and administer the correct dose and route of Naloxone within five minutes of recognizing an opioid overdose, 3) perform at least two basic life support (BLS) interventions, such as airway management and bag-valve mask ventilation, 4) communicate effectively with team members by providing clear instructions and patient status updates at least three times during the simulation.

**Educational Methods:**

In this study, high-fidelity simulation was implemented by creating a patient scenario of an opioid overdose, where students were required to recognize the symptoms and administer appropriate treatment, specifically Naloxone. The simulation was a component of the third-year emergency medicine clerkship curriculum.

**Research Methods:**

Learners completed pre- and post-simulation surveys assessing confidence in recognizing and managing opioid overdose, administering Naloxone, and performing airway interventions. The surveys used 5-point Likert scales to evaluate perceived competence and simulation effectiveness.

**Results:**

The simulation significantly improved learners’ confidence and knowledge in recognizing, managing, and treating opioid overdoses. Post-simulation surveys demonstrated marked gains across all domains of assessment, confirming the educational effectiveness of the scenario.

**Discussion:**

Overall, the educational content was highly effective. The significant increase in students’ confidence and knowledge regarding the recognition and treatment of opioid overdoses demonstrates that the hands-on, high-fidelity simulation successfully met its objectives. By immersing students in a realistic scenario and allowing them to practice administering Naloxone, the simulation prepared them to handle real-life cases with greater confidence and competence.

From its implementation, we learned that simulation-based education is a powerful tool for teaching critical skills in emergency medicine, particularly for life-threatening situations like opioid overdose. The overwhelmingly positive feedback from students further reinforced that they found the simulation valuable and informative.

**Topics:**

Opioid overdose recognition and treatment, emergency medicine education, high-fidelity simulation, Naloxone administration, recognition of overdose symptoms, treatment of opioid overdose, clinical confidence, patient-simulated experience, opioid epidemic, mu-opioid receptor antagonism, knowledge and confidence assessed via pre- and post-simulation surveys.

## USER GUIDE

**Table:** 

List of Resources:	
Abstract	1
User Guide	3
Instructor Materials	5
Operator Materials	14
Debriefing and Evaluation Pearls	18
Simulation Assessment	21
Appendix A: Assessment Tools and Survey Instruments	26

**Learner Audience:**

Medical Students

**Time Required for Implementation:**

**Preparation:** 60 minutes

**Time for case:** 20 minutes

**Time for debriefing:** 40 minutes

**Recommended Number of Learners per Instructor:**

3–5 learners per case. This allows for each student to engage directly with different roles, collaborative learning, and effective debriefing.

**Topics:**

Opioid overdose recognition and treatment, emergency medicine education, high-fidelity simulation, Naloxone administration, recognition of overdose symptoms, treatment of opioid overdose, clinical confidence, patient-simulated experience, opioid epidemic, mu-opioid receptor antagonism, knowledge and confidence assessed via pre-and post-simulation surveys.

**Objectives:**

By the end of the simulation session, learners will be able to:

1. Accurately identify the three key clinical signs of opioid overdose (respiratory depression, pinpoint pupils, unresponsiveness).
2. Identify and administer the correct dose and route of Naloxone within five minutes of recognizing an opioid overdose.
3. Perform at least two basic life support (BLS) interventions, such as airway management and bag-mask ventilation.
4. Communicate effectively with team members by providing clear instructions and patient status updates at least three times during the simulation.

### Linked objectives and methods

The goals and objectives of this opioid overdose simulation are achieved through a structured, scenario-based learning experience that mirrors real emergency department management of an unresponsive patient. The simulation provides learners with opportunities to recognize opioid toxicity, initiate evidence-based treatment, perform essential life support measures and communicate effectively within a clinical team.

Each educational objective is directly embedded in the case structure. Learners first encounter a patient presenting with respiratory depression, pinpoint pupils, and unresponsiveness— addressing the objective of identifying key clinical signs of opioid overdose. As the scenario progresses, learners must administer the correct dose and route of Naloxone within the expected time frame, demonstrating pharmacologic understanding and procedural efficiency. In situations where the patient’s response is delayed, learners are challenged to perform appropriate airway and ventilation techniques, reinforcing BLS competency.

Throughout the simulation, team members are expected to communicate clearly and effectively, using closed-loop communication and periodic updates to simulate real-world emergency coordination. The structured debrief following the scenario reinforces these objectives by prompting learners to analyze their decision-making, reflect on communication strategies, and discuss dosing challenges in fentanyl-resistant cases.

This approach ensures that each objective is met through active engagement with the case rather than theoretical discussion, making the simulation directly applicable to the emergency medicine environment and reproducible across other training programs.

### Recommended pre-reading for instructor

Recommend having familiarity with the guidelines for treating opiate overdose, the pharmacology of Naloxone, reviewing the best practices for high-fidelity simulation, and understanding an overview of the opioid epidemic. A recommended reading: Consensus Recommendations on the Treatment of Opioid Use Disorder in the Emergency Department. Hawk K, Hoppe J, Ketcham E, et al. *Ann Emerg Med*, Volume 78, Issue 3, 434–442.

### Learner responsible content

To keep the simulation experience as authentic as possible, avoiding any specific preparation on opioid overdose recognition and Naloxone use is ideal. The approach ensures that learners rely on their foundational medical knowledge, providing a more accurate assessment of their baseline skills and confidence levels.

### Results and tips for successful implementation

Before participating in the opioid overdose simulation, students completed a pre-simulation survey assessing baseline confidence in recognizing and managing opioid overdoses. Following the session, a post-simulation survey measured changes in confidence, perceived educational value, and overall effectiveness of the simulation. Each item was rated on a 5-point Likert scale (1 = Strongly Disagree to 5 = Strongly Agree).

Pre-Simulation Survey Questions

1. I received lectures in medical school regarding the pharmacology of opioids.
2. I received adequate education regarding the clinical presentation of opioid overdose.
3. I received adequate education regarding the treatment of opioid overdose.
4. I feel confident in recognizing the symptoms of opioid overdose.
5. I feel confident in treating a patient with opioid overdose.
6. I know which medication to administer in an opioid overdose.

Post-Simulation Survey Questions

1. The opioid overdose simulation was useful in my clinical education.
2. I learned something new about the clinical presentation of opioid overdose.
3. I learned something new about the treatment of opioid overdose.
4. I feel confident in recognizing the symptoms of opioid overdose.
5. I feel confident in treating a patient with opioid overdose.
6. I know which medication to administer in an opioid overdose.

Across 106 third-year medical students (102 with complete pre/post data), the simulation produced substantial increases in learner confidence and knowledge. Recognizing opioid overdose confidence rose from 67.9% to 97.0% agreement. Treating opioid overdose confidence improved from 34.0% to 89.2% agreement. Knowing the correct medication (Naloxone) agreement increased from 66.0% to 95.9%. Overall satisfaction: 97% found the simulation useful for their clinical education, and 99% reported learning something new about opioid overdose management. Learners described the simulation as realistic, practical, and directly applicable to their emergency medicine training. Open-ended feedback emphasized improved understanding of airway management, appropriate Naloxone dosing in fentanyl-resistant cases, and effective communication under high-stress conditions. Overall, participants reported that the scenario enhanced their clinical confidence and reinforced key skills essential to emergency practice.

**Table:** **Summary of Learner Confidence Changes:** Pre- and Post Simulation Survey Data

	Pre (%) Agree/Strongly Agree	Post (%) Agree/Strongly Agree
Recognizing opioid overdose	67.9%	97.0%
Treating overdose	34.0%	89.2%
Knowing correct medication	66.0%	95.9%

## Figures and Tables

**Table:** 

**#1**	**Physical Exam Cues on the Mannequin**: The high-fidelity mannequin should exhibit key physical signs of opioid overdose, including shallow breathing or apnea, cyanosis (bluish tint on the lips or fingertips, if possible with the mannequin), and pinpoint pupils. If the simulator allows a lack of response to verbal and physical stimuli, it can also be integrated to reinforce the severity of the overdose.
**#2**	**Handoff from EMS Provider**: A verbal handoff report provided by an EP in the role of EMS, detailing the patient’s presentation on scene, any interventions performed (such as blood sugar and vital signs), and any other available history.
**#3**	**Phone Call with Family Member**: A phone call stimulus from a family member who can provide background information on the patient’s recent opioid use, known allergies, or other pertinent history.
**#4**	**Simulated Naloxone Response**: If the mannequin has physiological feedback capabilities, adjust the vital signs to simulate an improvement in respiratory rate and consciousness shortly after Naloxone administration. This provides immediate feedback to the learners and reinforces the impact of their intervention.
**#5**	**Vital Signs Monitor**: Display fluctuating vital signs indicative of opioid overdose, such as low respiratory rate, decreased oxygen saturation, and low heart rate. The monitor can be adjusted to reflect changes based on learners’ interventions, such as an increase in oxygen saturation following successful airway management or Naloxone administration.
**#6**	**EKG:** showing sinus tachycardia; author’s own image
**#7**	**Chest radiograph:** showing a normal chest radiograph; author’s own image
**#8**	**Laboratory values:** including CBC, BMP, and urinalysis
**#9**	**PowerPoint**: includes triage information, initial vital signs, EKG, chest radiograph, and laboratory findings.

**Table:** 

**Vitals:**	Heart Rate (HR) 110	Blood Pressure (BP) 80/45
	Respiratory Rate (RR) 4	Temperature (T) 36.4°C
	Oxygen Saturation (O_2_Sat) 89%	

**Figure:**
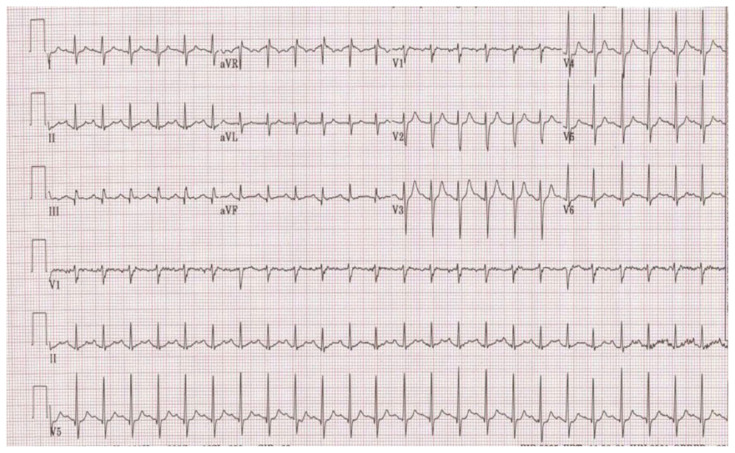


**Figure:**
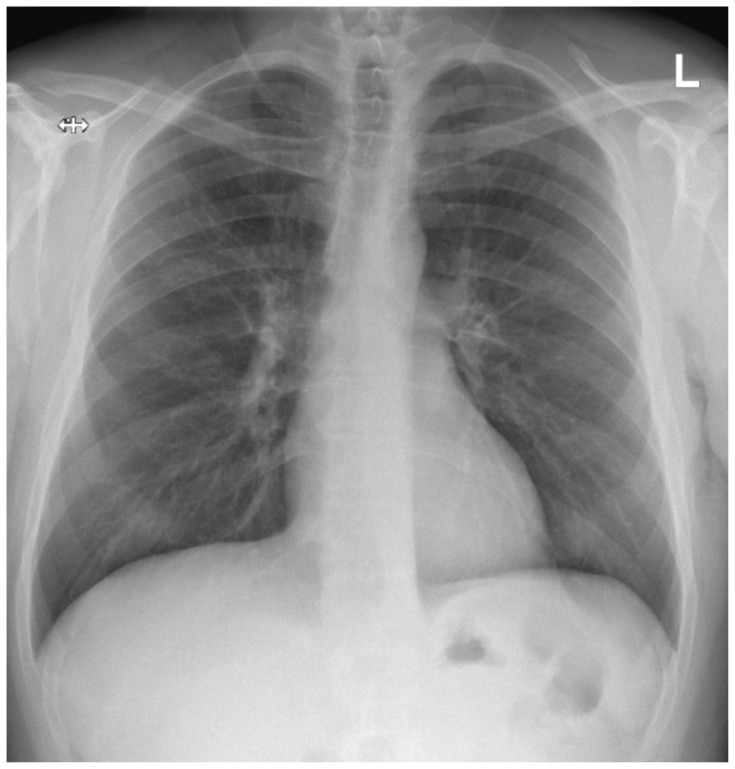


**Table:** 

*CBC (Complete Blood Count)*	
White blood count (WBC)	15.2 × 1000/mm^3^
Hemoglobin (Hgb)	13.0 g/dL
Hematocrit (HCT)	40%
Platelet (Plt)	420 × 1000/mm^3^
Differential	
Segs	70%
Bands	1%
Lymphs	24%
Monos	4%
Eos	1%

**Table:** 

*Basic metabolic panel (BMP)*	
Sodium	143 mEq/L
Potassium	4.2 mEq/L
Chloride	109 mEq/L
Bicarbonate (HCO3)	16 mEq/L
Blood Urea Nitrogen (BUN)	16 mg/dL
Creatinine (Cr)	0.9 mg/dL
Glucose	115 mg/dL

**Table:** 

*Urinalysis*	
Color	yellow, clear
Specific Gravity	1.015
Glucose	negative
Protein	negative
Ketones	negative
Leukocyte esterase	negative
Nitrites	negative
RBCs	0
WBCs	0

**Table:** SIMULATION EVENTS TABLE

Minute (state)	Participant action/trigger	Patient status (simulator response) & operator prompts	Monitor display (vital signs)
0:00 (Baseline)	Learners receive initial handoff from EMS	Patient is unresponsive, breathing shallowly, with pinpoint pupils, cool/pale skin. “EMS reports patient found unresponsive at home with a blood glucose of 110.”	T: 36.4 °C HR: 110 BP: 80/45 RR: 4 O2: 89% RA
0:03	Rapid assessment of ABCs, with initiation of basic airway interventions	Patient remains unresponsive and minimally responsive to painful stimuli (moans on sternal rub). Oxygen saturation increases slightly if BVM used. Prompt minimal moaning sound in response to painful stimuli.	HR: 110 BP: 80/45 RR: 4 O2 increases to 92% if BVM used
0:03 (Airway Management Branch Point)	Learners decide whether to initiate bag-mask ventilation (BVM) immediately or delay while continuing assessment	If BVM initiated promptly, oxygen saturation improves from 89% to 96% and heart rate decreases slightly as perfusion improves. Prompt: “You notice the patient’s oxygen saturation increasing.” If airway management is delayed, oxygen saturation drops to 82%, and the patient becomes more cyanotic with shallow respirations. Prompt: “The monitor shows decreasing oxygen saturation.”	Early BVM: HR: 100 BP: 90/50 RR: 6 O2: 96% Delayed Airway: HR: 120 BP: 85/45 RR: 4 O2: 82%
0:05 (Naloxone Branch Point)	Learners identify opioid overdose as potential cause and prepare Naloxone	If Naloxone is not administered, there is no change in patient status. Nurse prompt: “Are there other interventions you would consider?” If the recommended 0.4 mg dose is given, patient’s respiratory rate increases gradually, with mild improvement in responsiveness. Prompt: “You notice an increase in respiratory rate and slight responsiveness.”	Naloxone not given: HR: 110 BP: 80/45 RR: 4 O2: 89% Naloxone 0.4mg given: HR: 105 BP: 90/50 RR: 10 O2: 95%
0:06 (If 2 mg Naloxone given)	Learners administer 2 mg of Naloxone	Patient wakes abruptly, becomes combative and belligerent, vomiting and yelling, “What’s going on? Get me out of here!” Instructor emphasizes airway protection, communication with nursing staff, and de-escalation techniques.	Naloxone 2 mg given: HR: 130 BP: 160/90 RR: 16 O2: 96%
0:10	Learners reassess ABCs, monitor vital signs, and administer IV fluids for hypotension	With recommended dosing, patient opens eyes slightly and responds minimally to voice, able to speak weakly if prompted. Prompt slight eye opening and weak response to verbal stimuli. Blood pressure stabilizes after fluid administration. Prompt: “The patient’s blood pressure seems to be improving with fluid.”	HR: 95 BP: 105/60 RR: 12 O2: 98%
0:11 (Family Communication)	Learners contact the patient’s family to provide an update on the patient’s status and treatment.	Nurse prompt: “The patient’s mother has arrived in the waiting room asking for an update.” Learners summarize events, discuss the patient’s improvement, and address family concerns empathetically. Instructor reinforces use of clear, compassionate language.	HR: 95 BP: 105/60 RR: 12 O2: 98%
0:13 (Disposition Discussion)	Learners ask about recent drug use and discuss opioid addiction treatment options with patient	Patient becomes coherent, admits to using heroin after prescribed pain medication was discontinued. Patient says, “I was using pain pills, but my doctor stopped them, so I started buying stuff off the street.” Learners discuss continued monitoring for recurrent respiratory depression, consideration of Naloxone infusion if symptoms recur, and admission to an observation or monitored unit for at least 2 hours. They also address post-overdose counseling, addiction treatment referral, and harm-reduction strategies prior to discharge. Instructor reinforces evidence-based post-resuscitation management.	Stable BP and vital signs maintained
0:17 (Case Completion/Debrief Transition)	Learners summarize care and communicate disposition plan to team and instructor	Simulation ends with patient stable, responsive, and engaging in discussion about addiction treatment options. Operator initiates debrief with summary of actions taken.	HR: 95 BP: 110/65 RR: 14 O2: 98%

**Table:** 

**Critical Actions:** Rapid Assessment of ABCs Airway management and respiratory support with oxygen and BVM Identification of opioid overdose as the primary cause Naloxone administration, with an appropriate dose and management of any potential adverse reactions. IV fluid administration to manage hypotension	0:00

**Table:** 

	Milestone	Did not achieve level 1	Level 1	Level 2	Level 3
1	**Emergency Stabilization (PC1)**	□ Did not achieve Level 1	□ Recognizes abnormal vital signs	□ Recognizes an unstable patient, requiring intervention Performs primary assessment Discerns data to formulate a diagnostic impression/plan	□ Manages and prioritizes critical actions in a critically ill patient Reassesses after implementing a stabilizing intervention
2	**Performance of focused history and physical (PC2)**	□ Did not achieve Level 1	□ Performs a reliable, comprehensive history and physical exam	□ Performs and communicates a focused history and physical exam based on chief complaint and urgent issues	□ Prioritizes essential components of history and physical exam given dynamic circumstances
3	**Diagnostic studies (PC3)**	□ Did not achieve Level 1	□ Determines the necessity of diagnostic studies	□ Orders appropriate diagnostic studies. Performs appropriate bedside diagnostic studies/procedures	□ Prioritizes essential testing Interprets results of diagnostic studies Reviews risks, benefits, contraindications, and alternatives to a diagnostic study or procedure
4	**Diagnosis (PC4)**	□ Did not achieve Level 1	□ Considers a list of potential diagnoses	□ Considers an appropriate list of potential diagnosis May or may not make correct diagnosis	□ Makes the appropriate diagnosis Considers other potential diagnoses, avoiding premature closure
5	**Pharmacotherapy (PC5)**	□ Did not achieve Level 1	□ Asks patient for drug allergies	□ Selects an medication for therapeutic intervention, consider potential adverse effects	□ Selects the most appropriate medication and understands mechanism of action, effect, and potential side effects Considers and recognizes drug-drug interactions
6	**Observation and reassessment (PC6)**	□ Did not achieve Level 1	□ Reevaluates patient at least one time during case	□ Reevaluates patient after most therapeutic interventions	□ Consistently evaluates the effectiveness of therapies at appropriate intervals
7	**Disposition (PC7)**	□ Did not achieve Level 1	□ Appropriately selects whether to admit or discharge the patient	□ Appropriately selects whether to admit or discharge Involves the expertise of some of the appropriate specialists	□ Educates the patient appropriately about their disposition Assigns patient to an appropriate level of care (ICU/Tele/Floor) Involves expertise of all appropriate specialists
9	**General Approach to Procedures (PC9)**	□ Did not achieve Level 1	□ Identifies pertinent anatomy and physiology for a procedure Uses appropriate Universal Precautions	□ Obtains informed consent Knows indications, contraindications, anatomic landmarks, equipment, anesthetic and procedural technique, and potential complications for common ED procedures	□ Determines a back-up strategy if initial attempts are unsuccessful Correctly interprets results of diagnostic procedure
20	**Professional Values (PROF1)**	□ Did not achieve Level 1	□ Demonstrates caring, honest behavior	□ Exhibits compassion, respect, sensitivity and responsiveness	□ Develops alternative care plans when patients’ personal beliefs and decisions preclude standard care
22	**Patient centered communication (ICS1)**	□ Did not achieve level 1	□ Establishes rapport and demonstrates empathy to patient (and family) Listens effectively	□ Elicits patient’s reason for seeking health care	□ Manages patient expectations in a manner that minimizes potential for stress, conflict, and misunderstanding. Effectively communicates with vulnerable populations, (at risk patients and families)
23	**Team management (ICS2)**	□ Did not achieve level 1	□ Recognizes other members of the patient care team during case (nurse, techs)	□ Communicates pertinent information to other healthcare colleagues	□ Communicates a clear, succinct, and appropriate handoff with specialists and other colleagues Communicates effectively with ancillary staff

## INSTRUCTOR MATERIALS

**Case Title:** Opioid Overdose Simulation in Medical Student Education

**Case Description & Diagnosis (short synopsis):** The simulation case involves a patient experiencing an opioid overdose, a common and life-threatening emergency. The patient presents with signs of opioid intoxication, including respiratory depression, pinpoint pupils, and decreased responsiveness. The objective for learners is to recognize these symptoms, make a prompt diagnosis of opioid overdose, and administer appropriate treatment— primarily the use of Naloxone to reverse the overdose effects.

**Equipment or Props Needed:**

Essential equipment and props include a high-fidelity simulator mannequin capable of displaying respiratory depression, decreased responsiveness, and other physiological signs consistent with an opioid overdose. A vital signs monitor is necessary to display real-time metrics such as heart rate, respiratory rate, oxygen saturation, and blood pressure, which can be adjusted to reflect the patient’s condition and response to treatment. Additionally, a simulated Naloxone kit (either intranasal, intravenous, or intramuscular) should be available for learners to practice the correct administration techniques. Airway management supplies, including a bag-valve mask (BVM), oxygen mask, and airway adjuncts like an oropharyngeal airway, are also essential to simulate appropriate resuscitative interventions. Advanced airway equipment (endotracheal intubation) may also be necessary. An IV pole and supplies can be included for intravenous access allowing learners to practice IV setup and fluid administration. Medication labels and syringes (if no Naloxone kit) can be used for simulated medication preparation and dosing, further enhancing the realism of the scenario and enabling learners to develop practical skills in managing an opioid overdose.

**Embedded participants needed:**

1. **Nurse**: An assistant to play the role of a nurse, assisting the learners with tasks like administering oxygen, obtaining vital signs, and responding to team instructions. This role also allows learners to practice delegation and effective communication within a healthcare team.
2. **EMS Provider (optional)**: An assistant to portray the EMS provider who brought the patient to the emergency department. They can provide a brief handoff report to learners at the start of the simulation, including details about the patient’s condition upon arrival and any interventions performed in the field.
3. **Family Member (by phone)**: A confederate acting as a family member or friend, available by phone to provide additional history or background on the patient, such as recent opioid use or other relevant social information. This adds complexity to the scenario and allows learners to practice gathering collateral information and managing emotional responses.

**Stimulus Inventory:**

**Background and brief information:** The patient is a 22-year-old male brought to an academic medical facility by ambulance after being found unresponsive at home by his parents. His mother reported to EMS that he was unresponsive, with no recent medical interventions. On EMS arrival, the patient was unresponsive but had a normal blood glucose level of 110 mg/dL.

**Initial presentation:** Upon arrival in the emergency department, the patient remains unresponsive with vital signs indicating severe respiratory depression and hypotension. His initial examination shows a blood pressure of 80/45 mmHg, heart rate of 110 bpm, respirations at 4 breaths per minute, and oxygen saturation of 89% on room air. He appears pale and cool, with pinpoint pupils (1 millimeter, nonreactive) and shallow breaths. Physical examination reveals track marks on both arms, and a general unresponsive state, only exhibiting minimal response to painful stimuli.

**How the scenario unfolds: Initial State**: The patient arrives unresponsive with severe bradypnea, hypotension, and hypoxia.

**First Intervention (Airway and Breathing)**: Learners should perform a rapid assessment and recognize the need for airway management and apply supplemental oxygen and initiate ventilatory support with a bag-valve mask (BVM).

**Identification and Treatment of Opioid Overdose**: Then, the learners should administer Naloxone. The instructor can modulate the patient’s response based on the dose given:

- **0.4 mg Naloxone**: This low dose results in a gradual improvement, with increased respirations and the patient becoming more alert but cooperative, allowing for further engagement and discussion.
- **2 mg Naloxone**: A higher dose leads to a rapid awakening, with the patient becoming belligerent and combative. This response simulates the sudden withdrawal symptoms that can occur with a larger dose of Naloxone in opioid-dependent patients, challenging learners to manage an agitated patient safely.

**Response to Additional Actions**: If learners provide intravenous fluids, this can help stabilize the patient’s blood pressure and improve perfusion, which may prevent further complications.

The instructor should be prepared with:

- **History from the family:** Knowledge that the family suspects the patient was using street drugs for chronic pain management, which can be provided to learners if they initiate a conversation with family members by phone.
- **Progression of Symptoms if No Action is Taken**: If learners do not administer Naloxone or do not recognize the need for respiratory support, the patient’s condition should deteriorate, simulating a real-world consequence of delayed intervention.

**Case Outcome Based on Learner Actions**

1. **Appropriate Actions Taken**: If learners recognize the signs of opioid overdose, provide airway support, administer an appropriate dose of Naloxone, and stabilize the patient with oxygen and IV fluids, the scenario should progress smoothly, and the patient’s condition will improve. The patient becomes responsive and can engage in a brief discussion about opioid addiction, allowing learners to address the social and behavioral health aspects of the case.
2. **Inappropriate or Delayed Actions**: If the learners fail to recognize the opioid overdose or do not administer Naloxone in time, the patient’s condition will deteriorate, leading to worsening hypoxia, progressing to respiratory arrest, and eventually cardiac arrest if no intervention is made. This outcome reinforces the importance of timely action and provides a critical teaching moment in the debriefing session. Additionally, if the learners administer a high dose of Naloxone (eg, 2 mg or higher), it may precipitate acute withdrawal, resulting in a combative and agitated patient. This situation will require learners to manage a challenging behavioral response.

**Critical actions:**

1. Rapid assessment of ABCs.
2. Airway management and respiratory support with oxygen and BVM.
3. Identification of opioid overdose as the primary cause.
4. Naloxone administration, with an appropriate dose and management of any potential adverse reactions.
5. IV fluid administration to manage hypotension.

**Case Title:** Opioid Overdose Simulation in Medical Student Education

**Chief Complaint:** A 22-year-old male was found unresponsive at his home by his mother and father. His mother called 911, stating that the patient was unresponsive. On arrival of EMS, the patient is unresponsive with a blood sugar of 110.

**General Appearance:**

**Primary Survey:**

- **Airway:** Clear and patent
- **Breathing:** Shallow and slow
- **Circulation:** Palpable tachycardic peripheral pulses

**History:**

- **History of present illness:** The patient is a 22-year-old male who was found unresponsive at home by his mother and father. Initial history given by EMS who report that his mother called 911 after noticing that he was not waking up and was breathing abnormally. The family is available by phone and, if contacted, reveals that the patient’s doctor recently stopped prescribing him pain medications for his chronic back pain, and they suspect he may have been obtaining pain medications from the street.
- **Past medical history:** Chronic back pain
- **Past surgical history:** Unavailable; if contact parents, they state none
- **Patient’s medications:** No recent medication prescriptions
- **Allergies:** None
- **Social history:** Unavailable; if contact parents, they are unsure but suspect he may have been obtaining pain medications from the street
- **Family history:** Unavailable; if contact parents, they state no significant family history

**Secondary Survey/Physical Examination:**

- **General appearance:** Appears unresponsive and pale
- **HEENT:**
  ○ **Head:** wnl (within normal limits)
  ○ **Eyes:** 1 millimeter and non-reactive
  ○ **Ears:** wnl
  ○ **Nose:** wnl
  ○ **Throat:** wnl
- **Neck:** wnl
- **Heart:** tachycardia, regular rhythm
- **Lungs:** clear, shallow breathing
- **Abdominal/GI:** wnl
- **Genitourinary:** wnl
- **Rectal:** wnl
- **Extremities:** wounds in bilateral antecubital spaces consistent with track marks (need to specifically ask about an extremity exam, or if they attempt to place an IV in the antecubital space, the information is provided)
- **Back:** wnl
- **Neuro:** minimal moans to vigorous sternal rub, no eye-opening
- **Skin:** pale, cool
- **Lymph:** wnl
- **Psych:** wnl, however, if high-dose Naloxone is given, the patient becomes belligerent and agitated.

*EKG*

Author’s own image

*Chest Radiograph*

Author’s own image

Laboratory Results:

## OPERATOR MATERIALS

**Diagnosis:**

Opioid Overdose

**Disposition:**

The appropriate disposition for this patient, after successful resuscitation and stabilization, is either prolonged observation in the emergency department or admission to the hospital for further monitoring. Given the severity of the opioid overdose and the risk of recurrent respiratory depression, the patient should be observed in a setting with continuous monitoring capabilities. Prolonged observation in the emergency department may be appropriate if the patient’s condition improves significantly following Naloxone administration and inpatient resources are limited. This allows for close monitoring of respiratory status to ensure there is no recurrence of respiratory depression as the effects of Naloxone wear off. However, if the patient remains unstable or has persistent respiratory depression, admission to an ICU or step-down unit is preferred, providing a more controlled environment for managing any withdrawal symptoms that may arise and for implementing a long-term treatment plan. Learners must recognize when the patient is clinically stable, communicate the disposition plan effectively to the team and family, and coordinate safe transfer or continued observation. These actions align directly with the objectives of applying critical decision-making, performing essential life support interventions, and demonstrating effective interprofessional communication. During debriefing, learners reflect on their rationale for disposition choice, reinforcing clinical judgment and teamwork under realistic emergency conditions.

In either setting, a multidisciplinary approach involving addiction medicine or social work is recommended to address the patient’s opioid use disorder. This includes discussing treatment options, connecting the patient with counseling services, and exploring the possibility of medication-assisted treatment if appropriate.

## DEBRIEFING AND EVALUATION PEARLS

### Opioid Overdose Simulation in Medical Student Education

**Pearls:** The learning points of this opioid overdose case focus on both the medical management of opioid toxicity and the broader context of addressing opioid addiction. Through this simulation, learners are expected to recognize classic signs of opioid overdose, such as respiratory depression, pinpoint pupils, unresponsiveness, and hypotension, and understand the importance of early identification to initiate life-saving interventions. Learners will also develop skills in airway and respiratory management, including providing supplemental oxygen and using a bag-valve mask if necessary to support breathing before administering Naloxone. Faculty should review the airway-first principle, highlighting guidelines that prioritize ventilation support before pharmacologic reversal in suspected opioid overdose.^3^ The simulation emphasizes appropriate Naloxone administration, teaching learners the indications, dosing, and potential effects of the antidote. They will understand the rationale for starting with a lower dose to avoid withdrawal symptoms in opioid-dependent patients and learn how to manage any agitation that may occur if a larger dose is used. Debriefing should include evidence-based dosing strategies and review of safety data concerning withdrawal and excessive dosing.^3^

Additionally, learners will address hemodynamic instability by recognizing and treating hypotension and poor perfusion with intravenous fluids. This case also emphasizes the importance of prolonged observation or hospital admission due to the risk of recurrent respiratory depression after Naloxone administration. Learners will evaluate disposition options, understanding when prolonged emergency department observation or inpatient admission is most appropriate based on the patient’s condition.

In many real-world cases, emergency medical services (EMS) will have already administered Naloxone before ED arrival. Learners should consider this when evaluating persistent respiratory depression or minimal response. In the setting of high-potency synthetic opioids such as fentanyl, higher or repeated doses of Naloxone may be required. Discussion should emphasize reassessment after each dose, escalation from 0.4 mg to 2 mg (or higher) as indicated, and preparation for airway support if response remains incomplete. This reinforces clinical realism and dosing considerations frequently encountered in emergency practice. Beyond the acute management, this case teaches learners to address the patient’s underlying opioid use disorder with a compassionate, holistic approach. They will practice discussing opioid addiction, treatment options, and available resources, such as counseling and medication-assisted treatment, with the patient. Faculty should guide reflection on stigma, empathy, and patient-centered communication, supported by literature on effective teamwork and communication frameworks such as TeamSTEPPS.^4^

During debriefing, facilitators should prompt learners to analyze their decision-making process and team communication performance, acknowledging that structured debriefing is associated with improved learning outcomes in simulation-based medical education.^5^,^6^

**Other debriefing points:** The following are some debriefing tips specific to this opioid overdose simulation, based on our experience:

1. **Encourage Reflection on Missed History**: If learners did not engage EMS for further information or ask about contacting family, prompt them by asking, “Were there any other sources of history you could have solicited?” This encourages them to think about all possible avenues for gathering background information in similar cases.
2. **Discuss Naloxone Dosing Decisions**: If learners chose a high initial dose of Naloxone, and the patient became agitated, ask, “What considerations could guide your choice of Naloxone dosing?” This allows learners to reflect on the rationale for starting with a lower dose in opioid-dependent patients to avoid precipitated withdrawal.
3. **Address Airway and Ventilatory Support**: If learners delayed providing respiratory support or administering oxygen, discuss the importance of prioritizing airway management in opioid overdose cases. Ask, “How did your approach to airway and breathing impact the patient’s stability? What could be done differently?”
4. **Explore Decision-Making on Disposition**: If learners hesitated about whether to admit the patient or observe in the ED, guide them to discuss the pros and cons of each option based on the patient’s stability and the risk of recurrent respiratory depression. Ask, “What factors should you consider when deciding on observation vs. admission in a patient with opioid overdose?”
5. **Emphasize Compassionate Communication**: In the debrief, discuss the importance of a compassionate approach when addressing the patient’s opioid use disorder. If learners did not initiate a conversation about addiction treatment, you might ask, “How could you discuss long-term treatment options with this patient once he is stable?” This encourages learners to recognize the physician’s role in addressing addiction as part of holistic patient care.
6. **Reinforce Team Communication and Delegation**: If learners struggled with team roles or delegation, ask, “How could communication and teamwork have been improved during this scenario?” This encourages them to reflect on effective communication in high-stress scenarios and the importance of clearly defined roles.
7. **Use Open-Ended Questions to Facilitate Critical Thinking**: Ask questions like, “What other differential diagnoses could you consider in a case like this?” and “How did you prioritize interventions based on the patient’s initial presentation?” These questions help learners broaden their clinical reasoning and decision-making skills.

These debriefing tips encourage learners to reflect deeply on the case, consider alternative actions, and recognize both the technical and interpersonal skills necessary for managing opioid overdose patients effectively.

**Wrap Up:** There is no wrap-up lecture following the simulation. Additionally, there are no further references, suggestions for additional reading, or specific worksheets for observing learners. The debrief serves as the primary tool for reflection and discussion, focusing on key learning points and encouraging learners to review their performance and decision-making during the scenario.

## SIMULATION ASSESSMENT

*Opioid Overdose Simulation in Medical Student Education*

Learner: _________________________________________

***Assessment Timeline***

This timeline is to help observers assess their learners. It allows observer to make notes on when learners performed various tasks, which can help guide debriefing discussion.

**Critical Actions:**

□ Airway management and respiratory support with oxygen and BVM
□ Identification of opioid overdose as the primary cause
□ Naloxone administration, with an appropriate dose and management of any potential adverse reactions.
□ IV fluid administration to manage hypotension

**Summative and formative comments:**

**Milestones assessment:**

## INSTRUCTOR MATERIALS

### Appendix A Assessment Tools and Survey Instruments

#### Pre-Simulation Survey

**Table:**

1. I am confident in recognizing the signs and symptoms of an opioid overdose.				
Strongly Disagree	Disagree	Neutral	Agree	Strongly Agree
1	2	3	4	5
2. I am confident in treating a patient with an opioid overdose.				
Strongly Disagree	Disagree	Neutral	Agree	Strongly Agree
1	2	3	4	5
3. I know which medication to administer for an opioid overdose.				
Strongly Disagree	Disagree	Neutral	Agree	Strongly Agree
1	2	3	4	5
4. I understand the appropriate dose and route of Naloxone for an opioid overdose.				
Strongly Disagree	Disagree	Neutral	Agree	Strongly Agree
1	2	3	4	5
5. I am confident communicating effectively with a team during an opioid overdose scenario.				
Strongly Disagree	Disagree	Neutral	Agree	Strongly Agree
1	2	3	4	5
6. I feel prepared to manage an opioid overdose in the emergency department.				
Strongly Disagree	Disagree	Neutral	Agree	Strongly Agree
1	2	3	4	5

#### Post-Simulation Survey

**Table:**

1. I feel confident recognizing the signs and symptoms of an opioid overdose after completing the simulation.				
Strongly Disagree	Disagree	Neutral	Agree	Strongly Agree
1	2	3	4	5
2. I feel confident treating an opioid overdose after completing the simulation.				
Strongly Disagree	Disagree	Neutral	Agree	Strongly Agree
1	2	3	4	5
3. I know the correct medication (Naloxone) and dose to administer for an opioid overdose.				
Strongly Disagree	Disagree	Neutral	Agree	Strongly Agree
1	2	3	4	5
4. I can apply the skills learned in this simulation to real clinical scenarios.				
Strongly Disagree	Disagree	Neutral	Agree	Strongly Agree
1	2	3	4	5
5. I feel more confident communicating and working as part of a medical team during emergencies.				
Strongly Disagree	Disagree	Neutral	Agree	Strongly Agree
1	2	3	4	5
6. The simulation enhanced my understanding of opioid overdose management and was beneficial to my education.				
Strongly Disagree	Disagree	Neutral	Agree	Strongly Agree
1	2	3	4	5

#### Proposed Faculty Performance Assessment Tool (for Reproducibility)

Note: This tool was developed post hoc to provide a reproducible evaluation framework aligned with the educational objectives. It reflects the key domains faculty informally assessed during simulation observation.

**Table:**

Objective	Observable Behaviors/Assessment Criteria	Rating (1–5)
**Recognize opioid overdose**	Identifies respiratory depression, pinpoint pupils, and unresponsiveness	
**Administer Naloxone appropriately**	Chooses correct dose and route; re-evaluates patient response; avoids excessive dosing	
**Perform BLS interventions**	Initiates airway management and bag-valve ventilation effectively	
**Communicate effectively**	Uses clear, closed-loop communication and provides timely updates to team	

Faculty Comments:
